# A case–control study of innate immunity pathway gene polymorphisms in Puerto Ricans reveals association of toll-like receptor 2 +596 variant with asthma

**DOI:** 10.1186/s12890-016-0272-7

**Published:** 2016-08-05

**Authors:** Mario G. Ortiz-Martínez, Orquídea Frías-Belén, Sylvette Nazario-Jiménez, María López-Quintero, Rosa I. Rodríguez-Cotto, Braulio D. Jiménez-Vélez

**Affiliations:** 1 Department of Biochemistry, University of Puerto Rico-Medical Sciences Campus Main Bldg Lab B-210, San Juan, 00935 Puerto Rico USA; 2 Department of Biology, University of Puerto Rico at Humacao, Humacao, Puerto Rico USA; 3 Center for Environmental and Toxicological Research, University of Puerto Rico-Medical Sciences Campus, San Juan, Puerto Rico USA; 4 School of Public Health, University of Puerto Rico, Medical Sciences Campus, San Juan, Puerto Rico USA; 5 Department of Allergy and Immunology and School of Medicine Clinics, University of Puerto Rico-Medical Sciences Campus, San Juan, Puerto Rico USA; 6 Department of Internal Medicine and School of Medicine Clinics, University of Puerto Rico-Medical Sciences Campus, San Juan, 00935 Puerto Rico USA

**Keywords:** Asthma, Puerto Ricans, Toll-like receptors, Single nucleotide polymorphisms, Odds ratio, Genotyping, Toll-like receptor 2 +596 variant

## Abstract

**Background:**

For many years, African Dust Storms (ADE) has been thought to be associated with high prevalence of asthma in Puerto Rico (PR). Endotoxins (ENX) have been associated with ADE particulate matter (PM) and are known to promote pro-inflammatory responses in lung cells of susceptible individuals through the Toll-like receptor (TLR2/4) signaling pathways. Genetic variants are plausible contributors to such susceptibility. Therefore, we have evaluated a series of nine single nucleotide polymorphisms (SNPs) in TLR genes, which have been correlated positive and negatively to asthma prevalence and/or risk, in the Puerto Rican asthmatic population.

**Methods:**

The following SNPs were evaluated in 62 asthmatics and 61 controls through Taqman® Real Time PCR Assay: TLR4 (+896A/G, +1196C/T, −6687A/G); TLR2 (+596C/T, −16934 T/A, +399A/G, +1349C/T) and CD14 (−159C/T, +1188C/G). Genotypes were assessed for asthma association employing an odds ratio (OR) analysis.

**Results:**

Minor allele frequencies (*n* = 123) were determined for those variants as 0.07, 0.06, 0.35, 0.35, 0.37, 0.29, 0.04, 0.35 and 0.11, respectively. Two (+596C/T, +399A/G) TLR2 SNPs showed to be more represented in the asthmatic group by 89 % and 65 %, respectively. TLR4 SNP +896A/G analysis revealed only 1 G/G genotype (2 %) on the asthmatic group. The CD14 SNPs were similarly represented in the Puerto Rican population. Only the TLR2 +596 SNP was found to be significantly associated to asthma (*OR = 3.24 for CT, 2.71 for TT*) and particularly to females.

**Conclusions:**

The identification of TLR SNPs will reveal potential candidates for gene-environment interactions in Puerto Ricans. As far as we know this is the first study to evaluate this type of TLR gene polymorphisms in Puerto Rican asthmatics, contributing to the current knowledge in the Hispanic population.

**Electronic supplementary material:**

The online version of this article (doi:10.1186/s12890-016-0272-7) contains supplementary material, which is available to authorized users.

## Background

Asthma is an influential chronic illness in children and youth in the United States (US) and worldwide. Over the last decades it has been estimated that 300 million individuals are affected with asthma all over the world [1]. Asthma poses a heavy burden, which has steeply increased in minorities, particularly in Puerto Rican children [2]. Puerto Ricans exhibit the highest asthma prevalence (current: 16.6 % vs. 11.1 %/Non-Hispanic blacks, 8.2 %/Non-Hispanic whites and 6.3 %/total Hispanics for 2009 in US) than any other racial or ethnic group [3–6]. Recent statistics from the *National Health Interview Survey (NHIS)*, 2013 indicate that Puerto Ricans (18.8 %) had the higher asthma prevalence in the US; particularly in adult females (15.6 %). Not only in the island but also in the US, Puerto Ricans have high asthma prevalence [3] which suggests a genetic association. Ledogar et al. [7] found that Puerto Ricans living in New York City had asthma prevalence very similar to those living in the island (~16-18 %), especially pediatric patients from 0–12 yrs of age.

The striking racial and ethnic disparities in asthma prevalence cannot be solely attributed to one specific factor but to the interaction among many factors including genetic background, environmental exposures, nutrition, as well as socioeconomic status and access to health care [8, 9]. Indeed, genetic variation along with early-life environmental and lifestyle exposures correlated to admixture influences (African ancestry) can affect lung development and growth in Puerto Rican children [10]. A literature review for 2000–2009 revealed a list of 127 genes and respective proteins linked to asthma in which TLR pathway intermediaries are influential members [11]. Puerto Ricans are heavily exposed to PM airborne constituents such as ENX in constant African Dust Storms (ADE) [12–14]. Endotoxins are known ligands to Toll-like receptors (TLR2/4) and its co-receptor, the Cluster of Differentiation 14 (CD14) [15, 16]. The possible modification of ENX-receptor interaction and its link to specific single nucleotide polymorphisms (SNPs) could play significant role with asthma susceptibility [17]. Epidemiological studies have evaluated numerous polymorphisms in TLR2, TLR4 and CD14 in relation to asthma and similar phenotypes and some were significantly associated with high asthma prevalence, risk or severity. Two SNPs in the CD14 gene (−260C/T and -159C/T) have been shown to be associated with asthma susceptibility in French, Dutch and English populations [18–20]. The CD14 -159C/T variant was found to be as frequent as 0.67 in Puerto Ricans [21]. The TLR2 +596C/T variant was related to lower asthma risk in a French population [18]. Another group reports -16934 T/A to link significantly to a less tendency to have asthma diagnosis, symptoms and atopy between others [22]. Other TLR2 SNPs have been associated with allergic asthma [23] and TLR4 SNPs (Asp299Gly and T399I) with asthma pathogenesis in a German population [24]. Interestingly, the Asp299Gly SNP was found to be associated with 4-fold higher asthma prevalence and 7-fold higher prevalence of atopic asthma in a Swedish children population [25]. However, these findings have not been consistent in all studies [26]. Raby et al. [27] found no association of six different TLR4 SNPs and asthma susceptibility in three different ethnic groups from US and Canada. Furthermore, Nishimura et al. [28] observed no association between CD14 -159C/T SNP and asthma in a Japanese population. The variability in the association of the aforementioned SNPs to asthma outcomes may be attributed to inherent factors that are different among the populations studied and it has been suggested that gene-environment interactions may also explain the variable association [26, 29]. Choudry et al. [21] found association of the CD14 + 1437C/G SNP and asthma severity in Mexican and Puerto Rican asthmatics exposed to Environmental Tobacco Smoke, ETS and suggested a gene-environment interaction between ETS and that gene variant together with the -159C/T SNP. Exposure of Puerto Rican individuals with those variants showed a significant reduction in pulmonary function and immunoglobulin E (IgE) levels [21].

There has been no study performed on the Puerto Rican asthmatic population living in the island with particular interest in the frequency of polymorphisms in the TLR2/4 genes. This study evaluates specific SNPs in TLR2 (4 variants), TLR4 (3 variants) and CD14 (2 variants) genes in a Puerto Rican asthmatic population. The SNP selection was based on literature findings and their functionality in terms of significant associations to the condition of asthma.

## Methods

### Case–control study

Sixty-two Puerto Rican asthmatics 14 years old or more born from Puerto Rican parents and residents of Puerto Rico were recruited for the study. These subjects were diagnosed with asthma by a physician. The control group was represented by sixty-one Puerto Rican subjects without asthma, rhinosinusitis or any other chronic allergic and/or pro-inflammatory condition, including Chronic Obstructive Pulmonary Disease (COPD). A questionnaire containing personal, lifestyle, health, asthma family history, African Dust awareness and knowledge (perception) was completed by all subjects for descriptive and analytical purposes. Information about atopic status of subjects was not considered. Experimental and control subjects were recruited from the University of Puerto Rico, School of Medicine Clinics. Eligibility of all subjects was evaluated applying the inclusion/exclusion criteria established. Selected candidates were asked to sign an informed consent document, interviewed and asked to provide a saliva sample. The experimental design protocol (#A8570111) used in this study was reviewed and approved by the University of Puerto Rico – Medical Sciences Campus Institutional Review Board (UPR MSC IRB).

### DNA extraction and genotyping

Saliva samples were obtained from subjects using Saliva Collection Kits (SalivaGene® from B-Bridge International, Inc.). Genomic DNA extractions from these samples were performed following the manufacturer instructions. Extracted DNA was then diluted to 7 ng/μl and used for SNP genotyping assays through Real-Time Polymerase Chain Reaction (PCR) and Taqman® probes (Applied Biosystems) following the corresponding recommendations from the manufacturer. A total of 9 SNPs from three genes were analyzed. These were the TLR4 gene: +896A/G (rs4986790), +1196C/T (rs4986791) and -6687G/A (rs2737190); the TLR2 gene: +596C/T (rs3804099), −16934 T/A (rs4696480), +399A/G (rs13150331) and +1349 T/C (rs3804100); and in the CD14 gene: −159G/A (rs2569190), +1188C/G (rs4914).

### Statistical analyses

Three major parameters were used for sample size calculation: asthma prevalence (12.5-13 %, Continuous Health Survey for the Municipalities of Puerto Rico, 2003), the study site scenario of 696 patients (derived from 13–16 patients per week, where half or a third are asthmatics) and a confidence interval of 95 %. The *Epi-Info 6.04d software* was used as an instrument for that estimation, providing an approximate population size of 60 cases and 60 controls. Descriptive statistics (means, frequencies and standard deviations) were used to describe the studied population. Differences in the distribution of socio-demographic characteristics between asthmatics and controls were also evaluated. Multivariate analysis was performed to compare the following variables: 1) having asthma or not and 2) the presence or absence of one of the polymorphisms previously stated. Differences in proportions between groups were assessed by Pearson chi-square distribution, *t* test or Fisher’s exact test as appropriate. The magnitude of these associations was determined estimating prevalence odds ratio (OR) along with 95 % confidence intervals (CI) and p values for significance using a simple logistic regression model. Significant associations were adjusted by the potential confounding interaction variable of gender. Interaction terms formed by all combination pairs of the predictor variables within the multivariate model were tested with the likelihood ratio test. Data analysis was performed using the Data Analysis and Statistical Software STATA® 11.

Minor allele frequency (MAF) was calculated for each SNP in the experimental and control groups using the following equation: (N minor allele)/(total N × 2). Hardy-Weinberg equilibrium was tested through the *X*^*2*^ goodness-of-fit test to compare the observed SNP genotype frequencies with the expected ones. The Online Encyclopedia for Genetic Epidemiology Studies (OEGE) 2006-2011^©^ Hardy Weinberg calculator tool (www.oege.org/software/hardy-weinberg.html) was used to perform this test [30].

## Results

### Descriptive profiles

Sixty-two Puerto Rican asthmatics and sixty-one non-asthmatic control subjects were recruited for this case–control study. Table 1 illustrates the characteristic profiles for both study groups. The mean age for the asthmatic group was 41.2 ± 13.5 years and 46 ± 16.4 for the control. This particular characteristic was the only one that exhibited a statistically significant difference between the proportions of asthmatics and controls (p 0.035). Eighteen percent of the asthmatics were males while 82 % were females. In the controls, 34 % were males while 66 % were females. Most of the population studied lived in the metropolitan area, 64 % of asthmatics and 56 % controls. In terms of smoking habits, 31 % of the asthmatics and 23 % of the controls have smoke; and 83 % of the experimental group and 62 % of the control group were current smokers. A 54 % (asthmatics) vs. 51 % (controls) reported to have pets in their homes. Eighty-six percent of the asthmatics stated to have family history of asthma (first line relatives: parents and brothers/sisters) as compared to 81 % of the controls. A 48 % of the asthmatics reported to have experienced asthma exacerbations and the majority of them suffered the effects frequently. Eighty-nine percent of the asthmatics reported the use of asthma prescribed medications.

**Table 1:** Characteristic profiles of asthmatic and control subjects

Characteristic [N (%) or otherwise stated]	Puerto Rican^a^ Asthmatic Group	Puerto Rican Control Group
	*N = 62*	*N = 61*
Age in years [Mean (SD)]^d^	41.2 (13.5)	46 (16.4)
Age by Category		
14–24	5 (8.1)	3 (4.9)
25–35	20 (32.3)	20 (32.8)
36–45	13 (20.9)	5 (8.2)
46+	24 (38.7)	33 (54.1)
Gender [*N* (%) Males/*N* (%) Females]	11 (17.7)/51 (82.3)	21 (34.4)/40 (65.6)
Residence in Metropolitan (Urban) Area	40 (64.5)	34 (55.7)
Smoking^c^	19 (30.6)	14 (22.9)
Indoor Pets	33 (54.1)	31 (50.8)
Family Asthma History^b^	49 (86)	34 (80.9)
Asthma Exacerbations	30 (48.4)	
Asthma Medication Usage	55 (88.7)	
African Dust Knowledge	59 (95.2)	60 (98.4)
Asthma symptoms caused by African Dust	29 (46.8)	

*Legend*: ^a^Puerto Rican ancestry (European, West African and Native American); ^b^first line relatives: parents, sons (daughters) and/or brothers (sisters) have or had asthma; SD means standard deviation and ^c^the smoking characteristic is based on the answer to the question, have you ever smoked in your life? ^d^The only characteristic that showed a statistically significant difference between the proportions of asthmatics and controls was age with a *p* value of 0.035

As part of exposure to environmental events, the subjects were asked some questions about African Dust knowledge and perception. Almost all the subjects knew what the African Dust was: 95 % of the asthmatics and 98 % of the controls. Only, 47 % of the asthmatics experienced an adverse effect caused by the dust.

### SNP genotyping

Three TLR4, 4 TLR2 and 2 CD14 gene polymorphisms were evaluated in this study. The distribution of each SNP among asthmatics and controls is presented in Tables 2, 3 and 4. All gene variants were in Hardy Weinberg equilibrium as indicated by chi-square values at *p*-values >0.05 shown on Tables 2, 3 and 4.

**Table 2:** TLR4 SNP Genotypes and Minor Allele Frequencies (MAF) along with Hardy Weinberg Equilibrium Values in Asthmatic and Control Groups

SNP Genotypes	Asthmatics N (%)	Controls N (%)	Total N (%)	Hardy Weinberg Equilibrium (*X* ^*2*^, *p* > 0.05)
TLR4 +896				0.11
AA	54 (87.1)	51 (83.6)	105 (85.4)	
AG	7 (11.3)	10 (16.4)	17 (13.8)	
GG	1 (1.6)	0	1 (0.8)	
MAF (G)	0.06 (6)	0.08 (8)		
TLR4 +1196				0.52
CC	55 (88.7)	53 (86.9)	108 (87.8)	
CT	7 (11.3)	8 (13.1)	15 (12.2)	
MAF (T)	0.06 (6)	0.07 (7)		
TLR4 -6687				0.69
GG	10 (16.1)	11 (18.0)	21 (17.1)	
AG	36 (58.1)	29 (47.5)	65 (52.2)	
AA	16 (25.8)	21 (34.4)	37 (30.1)	
MAF (G)	0.37 (37)	0.33 (33)		

Hardy Weinberg equilibrium chi-square values were acquired under a *p*-value >0.05

**Table 3:** TLR2 SNP Genotypes and Minor Allele Frequencies (MAF) along with Hardy Weinberg Equilibrium Values in Asthmatic and Control Groups

SNP Genotypes	Asthmatics N (%)	Controls N (%)	Total N (%)	Hardy Weinberg Equilibrium (*X* ^*2*^, *p* > 0.05)
TLR2 +596				0.14
CC	7 (11.3)	17 (27.9)	24 (19.5)	
CT	36 (58.1)	27 (44.3)	63 (51.2)	
TT	19 (30.6)	17 (27.8)	36 (29.3)	
MAF (C)	0.35 (35)	0.36 (36)		
TLR2 -16934				0.65
TT	17 (27.4)	14 (23.0)	31 (25.2)	
AT	32 (51.6)	26 (42.6)	58 (47.2)	
AA	13 (21.0)	21 (34 .4)	34 (27.6)	
MAF (A)	0.35 (35)	0.38 (38)		
TLR2 +399				0.88
AA	23 (37.1)	29 (47.5)	52 (42.3)	
AG	33 (53.2)	26 (42.6)	59 (48.0)	
GG	6 (9.7)	6 (9.8)	12 (9.7)	
MAF (G)	0.32 (32)	0.26 (26)		
TLR2 +1349				2.53
TT	56 (90.3)	57 (93.4)	113 (91.9)	
CT	6 (9.7)	3 (4.9)	9 (7.3)	
CC	0	1 (1.6)	1 (0.8)	
MAF (C)	0.05 (5)	0.03 (3)		

Hardy Weinberg equilibrium chi-square values were acquired under a *p*-value >0.05

**Table 4:** CD14 SNP Genotypes and Minor Allele Frequencies (MAF) along with Hardy Weinberg Equilibrium Values in Asthmatic and Control Groups

SNP Genotypes	Asthmatics N (%)	Controls N (%)	Total N (%)	Hardy Weinberg Equilibrium (*X* ^*2*^, *p* > 0.05)
CD14 -159				0.05
GG	20 (32.2)	19 (31.2)	39 (31.7)	
AG	29 (46.8)	31 (50.8)	60 (48.8)	
AA	13 (21.0)	11 (18.0)	24 (19.5)	
MAF (A)	0.35 (35)	0.35 (35)		
CD14 +1188				1.72
CC	51 (82.3)	46 (75.4)	97 (78.9)	
CG	11 (17.7)	15 (24.6)	26 (21.4)	
MAF (G)	0.26 (26)	0.12 (12)		

Hardy Weinberg equilibrium chi-square values were acquired under a *p*-value >0.05

TLR4 +896 AA and AG genotypes were similar between the groups and only 1 homozygote GG was observed in the asthmatics. Minor allele frequencies were practically the same (Table 2). In the case of TLR4 +1196, the distribution of genotypes and MAF was similar in both groups. The TLR4 -6687 presented more AG genotypes in asthmatics, but more AA in controls. Minor allele frequency (G) was somewhat higher in asthmatics (37 % vs. 33 %).

TLR2 gene variant +596 showed more heterozygotes CT (58 %), more homozygotes TT (31 %) and less homozygotes CC (11 %) in asthmatics compared to controls (Table 3). Minor allele (C) was equally distributed in asthmatics and controls. Other 2 TLR2 variants, −16934 and +1349 revealed more heterozygotes in the asthmatic group, although more CC and AA homozygotes, respectively in the control group. The +1349 SNP showed only 1 CC homozygote in controls. Lastly, the +399 SNP presented more AG and less AA in asthmatics than among controls and the same GG in both groups. Minor allele frequency (G) was higher among asthmatics (32 % vs. 26 %).

The CD14 polymorphism, −159 was characterized by more AG genotypes in the control group and more AA genotypes in the asthmatic group (Table 4). The minor allele frequency (A) was the same in asthmatics and non-asthmatic controls. CD14 +1188 SNP was represented with more CG genotypes and higher MAF (G) in controls and more CC genotypes among asthmatics.

### Logistic regression and interaction analyses

A logistic regression analysis was performed to assess the association of each socio-demographic trait and having the condition of asthma (Table 5). Gender, age, education, marital status, residence, lifestyle (smoking and having pets) and asthma history were considered in the analysis. An OR of 2.43 resulted for the significant association of gender, specifically in females with asthma (p 0.038). Furthermore, asthma was associated to the age category of 36–45 years with an OR of 3.57 (p 0.031).

**Table 5:** Logistic Regression Analysis for the association of each socio-demographic trait and asthma. “Yes/No” categories were based on the answer of having or not the respective trait. Abbreviations: CI = confidence interval

Characteristics	OR	95 % CI	*P*-value
Gender			
Male	1.00		
Female	2.43	1.05-5.63	0.038
Age			
14-24	2.29	0.50-10.53	0.286
25-35	1.37	0.61-3.10	0.443
36-45	3.57	1.12-11.38	0.031
46+	1.00		
Education			
High School or less	2.00	0.67-5.99	0.216
Post-secondary Studies	1.22	0.45-3.28	0.694
Graduate Studies	1.00		
Marital Status			
Single	1.00		
Married/Living with someone	0.65	0.30-1.41	0.274
Widow	0.50	0.08-3.26	0.469
Divorced	0.64	0.09-2.20	0.481
Metropolitan Area Residence			
No	1.00		
Yes	1.44	0.70-2.98	0.321
Smoking Anytime in Life			
No	1.00		
Yes	1.48	0.66-3.32	0.337
Currently Smoking			
No	1.00		
Yes	3.13	0.59-16.58	0.181
Passive Smoking			
No	1.00		
Yes	1.50	0.50-4.52	0.471
First Line Asthmatic Relative			
No	1.00		
Yes	1.44	0.49-4.22	0.504
Indoor Pets			
No	1.00		
Yes	1.14	0.56-2.32	0.717

*Legend*: *OR* odds ratio, *CI* confidence interval

The analysis revealed a significant association between the TLR2 +596 CT genotype and asthma in the Puerto Rican population with an OR of 3.24, p 0.023 (Table 6). Moreover, the TT genotype was marginally associated to asthma with an OR of 2.71 (p 0.074). There was no association of any other TLR pathway SNPs with asthma.

**Table 6:** Logistic Regression Analysis for the association of each TLR4, TLR2 and CD14 SNPs and asthma

SNP Genotypes	OR	95 % CI	*P*-value
TLR4 +896			
AA	1.00		
AG	0.66	0.23-1.87	0.435
TLR4 +1196			
CC	1.00		
CT	0.84	0.29-2.49	0.757
TLR4 -6687			
GG	1.00		
AG	1.37	0.51-3.66	0.536
AA	0.84	0.29-2.46	0.748
TLR2 +596			
CC	1.00		
CT	3.24	1.18-8.91	0.023
TT	2.71	0.91-8.13	0.074
TLR2 -16934			
TT	1.00		
AT	1.01	0.42-2.43	0.976
AA	0.51	0.19-1.37	0.182
TLR2 +399			
AA	1.00		
AG	1.60	0.76-3.39	0.220
GG	1.26	0.36-4.43	0.718
TLR2 +1349			
TT	1.00		
CT	2.04	0.49-8.54	0.331
CD14 -159			
GG	1.00		
AG	0.89	0.49-2.00	0.774
AA	0.12	0.41-3.11	0.824
CD14 +1188			
CC	1.00		
CG	0.66	0.28-1.59	0.354
GG			

*Legend*: *OR* odds ratio, *CI* confidence interval

Analysis of the genotypes adjusted by gender also resulted in significant associations of TLR2 +596 genotypes and asthma in the Puerto Rican population (Table 7). Females with the CT genotype were strongly associated to asthma with an OR of 6.53 (p 0.004). The TT genotype, which was initially marginal in significance when using the crude OR analysis was statistically significant after the gender adjustment, where females exhibited an OR of 6.04 (p 0.008). Males with the CT genotype presented an OR of 0.67 but it was not significant (p 0.665). Another TLR2 SNP, −16934 turned to be significant in males with the AA genotype (OR 0.13/ p 0.039).

**Table 7:** Logistic Regression Analysis adjusted by gender of the association between TLR4, TLR2 and CD14 SNPs and asthma

	Males			Females		
	Adjusted OR	95 % CI	*P*-value	Adjusted OR	95 % CI	*P*-value
TLR4 +896						
AG	2.25	0.37-13.67	0.378	0.41	0.11-1.51	0.181
TLR4 +1196						
CT	3.56	0.50-25.56	0.206	0.48	0.13-1.84	0.286
TLR4 -6687						
AG	3.00	0.26-34.57	0.378	1.20	0.39-3.66	0.748
AA	0.33	0.02-7.14	0.482	1.11	0.33-3.75	0.865
TLR2 +596						
CT	0.67	0.11-4.17	0.665	6.53	1.82-23.40	0.004
TT				6.04	1.59-23.01	0.008
TLR2 -16934						
AT				2.40	0.85-6.73	0.096
AA	0.13	0.02-0.90	0.039	0.90	0.27-3.00	0.864
TLR2 +399						
AG	0.60	0.12-2.89	0.525	2.30	0.93-5.67	0.070
GG				0.74	0.18-3.05	0.678
TLR2 +1349						
CT				2.47	0.47-12.95	0.286
CD14 -159						
AG	1.94	0.29-13.19	0.496	0.73	0.29-1.85	0.503
AA	2.79	0.36-21.73	0.325	1.00	0.28-3.54	1
CD14 +1188						
CG				0.72	0.28-1.90	0.513

*Legend*: *OR* odds ratio, *CI* confidence interval

The likelihood ratio test revealed interaction between 2 of the 9 SNPs and asthma, TLR2 +596 (*X*^*2*^ = 10.21, p 0.0061) and TLR2 + 399 (*X*^*2*^ = 7.84, p 0.0199). Others did not present a significant interaction [TLR4 + 896 (*X*^*2*^ = 2.24, p 0.1348); TLR4 + 1196 (*X*^*2*^ = 2.77, p 0.0961); TLR4 -6687 (*X*^*2*^ = 3.66, p 0.1607) and CD14 -159 (*X*^*2*^ = 1.02, p 0.5999)].

## Discussion

The TLR2 +596 SNP (genotypes: CT and TT) was associated to asthma within the Puerto Rican population as demonstrated among French atopic subjects carrying the allele C, while non-atopic subjects revealed a positive but not significant association [18]. More importantly, the authors discovered a gene-environment interaction, where non-atopic subjects with the C allele were protected from asthma by means of living in a country environment. This variant was also studied in Korean patients of allergic rhinitis where no significant difference was found compared to controls and no correlation to total serum IgE levels, but it was associated to peripheral blood eosinophil counts [31]. Interestingly, MAFs (C) found in that study (cases: 0.32; controls: 0.31) correlate well with our findings in Puerto Rican asthmatics in spite of notable differences in sample size (cases: 0.35; controls: 0.36) [31, 32]. This polymorphism was associated to protection to non-atopic asthma in children from the International Study of Asthma and Allergy in Childhood (ISAAC) phase II [33]. A haplotype involving TLR +596 C and TLR2 +1349 C was also not related to allergic asthma in Norwegian asthmatic children [23].

The TLR2 +596 SNP was particularly associated to females after gender adjustments. The fact that this SNP was significantly predominant in Puerto Rican asthmatic females is consistent with a higher prevalence asthma reported for females vs. males: 20.4 vs. 12.7 by Akinbami et al. [6]. This marked difference can also be attributed to female steroid sex hormones. Bonds and Midoro-Horiuti [34] described estrogens involvement in allergic sensitization as well as lung function and mechanics, where for example they can relax airway smooth muscle and increase ciliary beat frequency. Sex hormones have been implicated in the interaction and modulation of TLR expression and function, where lipopolysaccharide-induced airway inflammation is reduced in female rats and increased after stimulation with testosterone [35, 36]. Other study has shown inhibition of TLR agonist-induced interleukin (IL-8) release by estradiol in cystic fibrosis bronchial epithelial cells [37].

The TLR2 +596 SNP is characterized as a synonymous or silent mutation (sSNP) whose functional mechanism is unknown. The concept of this type of SNPs of being “silent” is questionable since in some cases these mutations are responsible of more profound effects than non-synonymous SNPs and their importance relies on the comparable occurrence 1.1 % vs. 1.5 % for non-synonymous in coding regions [38]. Based on studies of other sSNPs it could create a translational pause or affect protein folding, mRNA splicing, mRNA structure and stability, protein abundance, microRNAs binding sites, co-translational protein folding and post-translational modifications via mRNA-RNA and mRNA protein interactions [38–42]. Speculations exist about how the +596 SNP could lower TLR2 expression in innate immune cell membranes [18, 43]. It has been hypothesized that this sSNP can alter function of the integrin β3 (ITGB3)-TLR2 complex, increasing mold sensitization; decrease cytokine production (TNF-α) in monocytes (CC genotype); increase (decrease in haplotype with other variants) TNF-α, IL-10 and IL-8 in peripheral blood leukocytes (T allele) stimulated with bacterial lipoprotein [43–45]. Finally, it has been suggested that TLR2 SNPs in its heterodimeric network is a major factor mediating inter-individual variation in in vitro cytokine responses to lipopeptide, Pam_3_CSK_4_ [33, 46].

The TLR2 SNP, +399A/G and TLR2 -16934 SNP (in males) also presented an interaction with asthma, but it was not considered important since the previous analysis did not reveal any significant associations. In support of this result, this SNP has not been associated with childhood asthma [47].

Our results for the TLR2, TLR4 and CD14 SNPs frequencies are similar to those that have been reported for Hispanic and Caucasian populations found in the National Center for Biotechnology Information (*NCBI)* SNP database (details about the comparison of the allele frequencies in the present Puerto Rican population with other racial groups included in Additional file 1: Table S1). Compared to Hispanics as a group, the +596 SNP presented more heterozygotes and TT homozygotes; but compared to Caucasians the frequency of TT was the same. Surprisingly, the TLR4 +1196 SNP frequencies obtained for the studied population are exactly the same as that found in Mexicans even though these two Hispanic groups present asthma disparities in terms of asthma prevalence, Puerto Ricans presenting the highest and Mexicans the lowest compared to non-Hispanic whites and blacks [4, 6]. The AA genotype of the TLR2 -16934 SNP was more representative in Puerto Ricans as compared to Caucasians and Hispanics. Frequencies for the TLR4 +896 and TLR2 +1349 SNP genotypes in Puerto Ricans are very similar to African Americans. Genotype frequencies for TLR4 +896, TLR2 +1349 and CD14 +1188 variants were somewhat similar to those reported for the African population. Frequencies for the CD14 SNP in asthmatics (AA = 0.32, AG = 0.47, GG = 0.21) relate well with that previously reported by Choudry, et al. [21] (0.29, 0.50 and 0.21, respectively). A significant association to asthma was not encountered possibly due to age differences between the two designs (childhood against adulthood). O’Donnell et al. [48] described an increased OR for atopy and airway hyper-responsiveness in children carriers of the CD14 -159CC genotype, while the association was not identified among adults, suggesting an age-dependent modulation of the atopic phenotype.

The analysis of genetic association to complex diseases (e.g. asthma) often exhibits result inconsistency. In fact, we found the TLR2 +596 SNP to be associated to asthma in Puerto Ricans, but others did not obtain any association with asthma in farmers/non-farmers European children [22]. Noguchi et al. [49] did not detect association to the development of asthma and total IgE serum levels in the presence of the +596 variant but stated that low frequency of some haplotypes including it as well as +1349 SNP impeded the relation to asthma development. These variable findings may be related to a number of factors which may represent limitations. Discrepancies in status of the risk/association of TLR variants in asthma can be explained by differences in ethnicity or admixture, environmental exposures such as dust allergens and endotoxins and even gene-gene and gene-environment interactions [50–57]. Low gene penetrance, low allele frequency and linkage disequilibrium are other factors that may avoid interactions of SNPs with disease [32, 33, 41, 50, 52, 58]. These properties probably explain why some of the other SNPs with relatively low MAF evaluated in our study did not result significant (TLR4 +896 and +1196 SNPs). Although a sample size calculation was performed, insufficient power [54, 59] could possibly prevent any link between the other SNPs and asthma. Replication of the presented genetic analysis is advised in an effort to account for those limitations and consider other unmeasured confounding factors.

Genetic variation in genes responsible for ENX recognition (TLRs) could modify their respective signaling pathway. Asthma susceptibility caused by this variation could be developed as an increase or reduction in NF-kB signaling that ultimately leads to an increase or reduction in the production of the pro-inflammatory mediators such as cytokines: interleukins IL-6 and IL-8. The expression of genetic variants can be modified by environmental effects of pollution, allergen exposure, social stressors and other conditions [60]. Interests in this aspect are seen in studies from Kerkhof et al. [61] where TLR2 -16934 (rs4696480) was shown to modify the effect of PM_2.5_ exposure (traffic-related) on doctor-diagnosed asthma prevalence in the age range of birth to 8 years in a population from Netherlands. The TLR2 +596 SNP was also evaluated too but did not interact with particle pollution. Nonetheless, increasing the sample size of the present study which is characterized by a different ethnic population could reveal a different biological scenario. Further studies are needed to confirm the functionality of this particular SNP in vitro.

## Conclusions

Our study demonstrates an association between the TLR2 +596 SNP and asthma in Puerto Ricans. This variant is a potential gene-environment interaction candidate that may be able to modify the biological effects generated by exposure to environmental endotoxins in particle pollution, which are known to exacerbate asthmatic symptoms, and in that way modulate negative or positively the pro-inflammatory response in asthmatic individuals. Overall, the study represents a step forward not only in searching causes to explain the characteristic high prevalence of asthma in the Puerto Rican population, but also in identifying SNPs that could be considered as targets for the advancement of the pharmacogenomics field. In the future, it will help in the improvement of current asthma therapy and will provide the basis for the effectiveness of personalized medicine in Puerto Rican asthmatics.

## Abbreviations

ADE, african dust storms or events; CI, confidence interval; COPD, chronic obstructive pulmonary disease; ENX, endotoxins; ETS, environmental tobacco smoke; ISAAC, international study of asthma and allergy in childhood; MAF, minor allele frequency; NCBI, national center for biotechnology information; NHIS, national health interview survey; OEGE, online encyclopedia for genetic epidemiology studies; OR, odds ratio; PCR, polymerase chain reaction; PM, particulate matter; PR, Puerto Rico; SNPs, single nucleotide polymorphisms; sSNP, silent single nucleotide polymorphism; TLR, toll-like receptor; US, United States

## Additional file

Additional file 1: Table S1. Comparison of TLR2, TLR4 and CD14 SNP frequencies among the analyzed Puerto Rican population with other racial groups – This table presents a comparison of the SNP genotype frequencies found in the studied Puerto Rican population (asthmatics and controls as one group) with other population heritages (Hispanic = HISP1; Caucasian = CAUC1; African American = AFR1; Mexican = HapMap MEX; Chinese = HapMap CHD; Japanese = HapMap JPT; Italian = HapMap TSI; African = HapMap YRI). The NCBI database was used as a source for those frequencies and ancestral alleles are highlighted. (DOC 59 kb)
