# Supplementary material for: A case–control study of innate immunity pathway gene polymorphisms in Puerto Ricans reveals association of toll-like receptor 2 +596 variant with asthma
Source: BMC Pulm Med. 2016 Aug 5;16:112. doi: 10.1186/s12890-016-0272-7 (PMC4974724; doi:10.1186/s12890-016-0272-7)
Supplement: Additional file 1: Table S1. — Comparison of TLR2, TLR4 and CD14 SNP frequencies among the analyzed Puerto Rican population with other racial groups – This table presents a comparison of the SNP genotype frequencies found in the studied Puerto Rican population (asthmatics and controls as one group) with other population heritages (Hispanic = HISP1; Caucasian = CAUC1; African American = AFR1; Mexican = HapMap MEX; Chinese = HapMap CHD; Japanese = HapMap JPT; Italian = HapMap TSI; African = HapMap YRI). The NCBI database was used as a source for those frequencies and ancestral alleles are highlighted. (DOC 59 kb) [file 12890_2016_272_MOESM1_ESM.doc]

**Supplementary Table 1**: *Comparison of TLR2, TLR4 and CD14 SNP frequencies among the analyzed Puerto Rican population with other racial groups.*

| **SNP Genotype** | **Population Heritage#** | | | | | | | | |
| --- | --- | --- | --- | --- | --- | --- | --- | --- | --- |
|  | **Puerto Rican^** | **Hispanic** | **Caucasian** | **African American** | **Mexican** | **Chinese** | **Japanese** | **Italian** | **African** |
| ***TLR4 +896*** | | | | | | | | | |
| A/A | 0.85 | 0.91 | 0.93 | 0.83 | 0.94 | 1.00 | 1.00 | 0.91 | 0.92 |
| A/G | 0.14 | 0.09 | 0.07 | 0.17 | 0.06 | - | - | 0.09 | 0.08 |
| G/G | 0.01 | - | - | - | - | - | - | - | - |
| ***TLR4 +1196*** | | | | | | | | | |
| C/C | 0.88 | 0.91 | 0.93 | 0.96 | 0.88 |  | 1.00 | 0.88 | 1.00 |
| C/T | 0.12 | 0.04 | 0.06 | 0.04 | 0.12 |  | - | 0.11 | - |
| T/T |  | 0.04 | - | - | - |  | - | - | - |
| ***TLR4 -6687*** | | | | | | | | | |
| G/G | 0.17 |  |  |  | 0.06 | 0.15 | 0.13 | 0.09 | 0.73 |
| A/G | 0.53 |  |  |  | 0.50 | 0.41 | 0.45 | 0.43 | 0.26 |
| A/A | 0.30 |  |  |  | 0.44 | 0.43 | 0.42 | 0.48 | 0.02 |
| ***TLR2 +596*** | | | | | | | | | |
| C/C | 0.20 | 0.09 | 0.13 | 0.33 | 0.10 | 0.02 | 0.07 | 0.19 | 0.42 |
| C/T | 0.51 | 0.48 | 0.58 | 0.50 | 0.50 | 0.56 | 0.40 | 0.48 | 0.42 |
| T/T | 0.29 | 0.43 | 0.29 | 0.17 | 0.40 | 0.42 | 0.53 | 0.33 | 0.16 |
| ***TLR2 -16934*** | | | | | | | | | |
| T/T | 0.26 | 0.35 | 0.26 | 0.71 |  |  | 1.00 | - | 1.00 |
| A/T | 0.46 | 0.43 | 0.55 | 0.25 |  |  | - | - | - |
| A/A | 0.28 | 0.22 | 0.19 | 0.04 |  |  | - | - | - |
| ***TLR2 +399*** | | | | | | | | | |
| A/A | 0.41 | 0.35 | 0.32 | 0.71 |  | 0.34 | 0.21 | 0.44 | 0.81 |
| A/G | 0.49 | 0.39 | 0.55 | 0.25 |  | 0.49 | 0.55 | 0.35 | 0.20 |
| G/G | 0.10 | 0.26 | 0.13 | 0.04 |  | 0.17 | 0.24 | 0.20 | - |
| ***TLR2 +1349*** | | | | | | | | | |
| T/T | 0.92 | 0.87 | 0.81 | 0.92 | 0.82 | 0.50 | 0.58 | 0.87 | 0.88 |
| T/C | 0.07 | 0.13 | 0.19 | 0.08 | 0.18 | 0.39 | 0.41 | 0.12 | 0.12 |
| C/C | 0.01 | - | - | - | - | 0.11 | 0.01 | - | - |
| ***CD14 -159*** | | | | | | | | | |
| G/G | 0.30 | 0.09 | 0.34 | 0.04 | 0.19 |  | 0.20 |  |  |
| A/G | 0.50 | 0.52 | 0.55 | 0.18 | 0.54 |  | 0.58 |  |  |
| A/A | 0.20 | 0.39 | 0.10 | 0.77 | 0.27 |  | 0.22 |  |  |
| ***CD14 +1188*** | | | | | | | | | |
| C/C | 0.79 | 0.74 | 0.93 | 0.80 |  |  | 0.96 |  | 0.85 |
| C/G | 0.21 | 0.26 | 0.07 | 0.21 |  |  | 0.04 |  | 0.15 |
| *G/G* |  | - | - | - |  |  | - |  | - |

Legend: ^Frequency in Puerto Ricans is presented as the complete (asthmatics + controls) population and based on the study reported here. The ancestral alleles are highlighted. ^#^National Center for Biotechnology Information (NCBI) SNP database was used as the primary reference to prepare this table. Hispanic = HISP1; Caucasian = CAUC1; African American = AFR1; Mexican = HapMap MEX; Chinese = HapMap CHD; Japanese = HapMap JPT; Italian = HapMap TSI; African = HapMap YRI
